# Impact of Sagittal Balance on Clinical Outcomes in Surgically Treated T12 and L1 Burst Fractures: Analysis of Long-Term Outcomes after Posterior-Only and Combined Posteroanterior Treatment

**DOI:** 10.1155/2017/1568258

**Published:** 2017-01-10

**Authors:** M. Mayer, R. Ortmaier, H. Koller, J. Koller, W. Hitzl, A. Auffarth, H. Resch, A. von Keudell

**Affiliations:** ^1^Spinal Surgery Center, Schoen Clinic Nuernberg-Fuerth, Europa-Allee 1, 90763 Fürth, Germany; ^2^Department for Traumatology and Sports Injuries, Paracelsus Medical University Salzburg, Muellner Hauptstrasse 48, 5020 Salzburg, Austria; ^3^Research Office and Biostatistics, Paracelsus Medical University Salzburg, Strubergasse 21, 5020 Salzburg, Austria; ^4^Department of Orthopaedic Surgery, Harvard Combined Orthopaedic Surgery Program, Massachusetts General Hospital, 55 Fruit Street, Boston, MA 02114, USA

## Abstract

*Objective*. Long-term radiological and clinical outcome retrospective study of surgical treatment for T12 and L1 burst fractures in perspective of sagittal balance measures.* Methods*. Patients with age of 16–60 years, complete radiographs, early surgical treatment surgery, and follow-up (F/U) > 18 months were included and strict exclusion criteria applied. Regional and thoracolumbar kyphosis angles (RKA and TLA) were measured preoperatively and at final F/U, as were parameters of the spinopelvic sagittal alignment. Clinical outcomes were assessed using validated measures.* Results*. 36 patients with age mean age of 39 years and F/U of 69 months were included. 61% of patients were treated with bisegmental posterior instrumentation (POST-I) and 39% with combined posteroanterior instrumented fusion (PA-F). At F/U, several indicators for clinical outcomes showed a significant correlation with radiographic measures in the overall cohort with inferior clinical outcomes corresponding with increasing residual deformity and sagittal malalignment. Statistical analysis failed to reach level of significance for the differences between POST-I and PA-F group at final F/U. Only a strong trend towards better restoration of the thoracolumbar alignment was observed for the PA-F group in terms of the RKA and TLA.* Conclusions*. Results in a surgically treated cohort of T12 and L1 burst fracture patients indicate that superior clinical outcomes depend on restoration of sagittal alignment.

## 1. Introduction

Thoracolumbar burst fractures account for up to 17% of major spinal fractures [1]. The majority of cases are the result of a fall from height, sports-related trauma, or a motor vehicle accident [2–4]. The appropriate management of thoracolumbar fractures without neurological deficits remains controversial. The heterogenetic characteristics of these fractures, fracture severity, associated injuries, comorbidities, social aspects, and legal demands have impeded conclusive comparative studies that compare nonsurgical with surgical treatment [5–10]. Previous studies reported comparable mid-term results for nonsurgical treatment with or without casting and for surgical intervention [1, 2, 11–14].

Aim of surgical treatment is to achieve stabilization of the injured thoracolumbar anatomy, early mobilization of the patient, restoration of physiological alignment, indirect decompression of the neural elements by segmental reduction maneuvers, and maintenance of reduction until bony union of the fracture and stabilization of the segments have occurred. Surgical options include instrumented fracture stabilization with or without fusion using a posterior, anterior, or combined posteroanterior approach [15–18]. Studies that examine the outcomes of burst fracture treatment using homogenous samples are limited and inconclusive regarding the superiority of clinical outcomes with either treatment strategy [19–22].

Nonsurgical treatment of burst fractures can result in posttraumatic kyphosis, which can ultimately lead to exaggerated compensatory mechanisms of the adjacent segments. These changes can also promote degenerative changes and pain [5]. With significant thoracolumbar kyphosis [23–25], sagittal imbalance will develop. Restoration of the spinal and spinopelvic sagittal alignment is becoming increasingly valued as an important prognostic indicator of improved clinical outcomes following deformity surgery [5, 26–28]. Therefore, the objective of the current study was to investigate the clinical and radiographic mid- to long-term results of surgically treated thoracolumbar burst fractures in a homogenous sample of patients with T12 or L1 burst fractures who met strict inclusion criteria. We sought to identify the factors that predict the radiographic and clinical outcomes of two standard surgical techniques according to measures of spinopelvic balance.

## 2. Materials and Methods

A case series review was performed on a consecutive series of patients with isolated T12 or L1 burst fractures collected from a prospective fracture database of a single trauma center. Patients were selected if they had been treated surgically using either posterior instrumentation (POST-I) only or combined posteroanterior instrumentation with anterior spinal fusion (PA-F). Inclusion criteria were absence of neurological deficits (Frankel E), minimum follow-up (FU) of 18 months, maximum delay between injury and surgical treatment of <10 days, and age ranging from 16 to 65 years at the time of injury. Informed consent was obtained from all patients included. Exclusion criteria were chronic musculoskeletal disease; specific spinal disorders and deformities, such as adult scoliosis, ankylosing spondylitis, thoracolumbar DISH, and rheumatoid arthritis; evidence of osteoporosis; previous thoracolumbar spine surgery; failure to complete follow-up at our institution; injuries to the lower extremities that would alter posture and ambulation; and the presence of a litigating worker's compensation claim.

Burst fractures were characterized using the AO Classification according to Magerl et al. [29] and the Load Sharing Classification (LSC) according to McCormack et al. [30]. Patients' records were examined with regard to demographics, surgical and hospital data, and complications. The complications were stratified according to Glassman et al. [31], and only major complications were recorded.

### 2.1. Surgical Technique and Postoperative Treatment

According to the attending surgeon in charge, patients were treated following the guidelines of the POST-I or PA-F group. As there is up to now no clear evidence of superiority of one over the other technique when treating AO Type 3 burst fractures, the decision of treatment was according to the attending surgeons' discretion. Decompression at the fracture level was conferred indirectly in all patients using closed reduction during posterior instrumentation.

**Figure 1:**
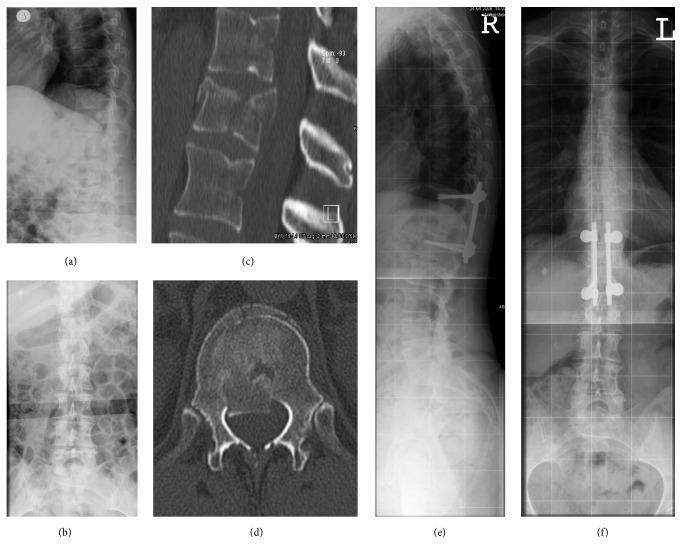
Illustrative case 1. A 62-year-old female patient with a T12 burst fracture after a fall while cross-country skiing (a, b). The fracture was classified as AO 3.2.1 and LSC 5 (c, d). Surgical treatment was performed using closed reduction and posterior instrumentation at T11–L1. The preoperative RKA and TLA were 5.1° and 11.2°, respectively, and the RKA and TLA at the final follow-up of 76 months postoperatively were 11.3° and 14°, respectively (e, f).

The patients in the POST-I group underwent an open bisegmental transpedicular screw fixation extending one level above to the vertebra below the burst fracture and sparing the fractured level (Figure 1).

**Figure 2:**
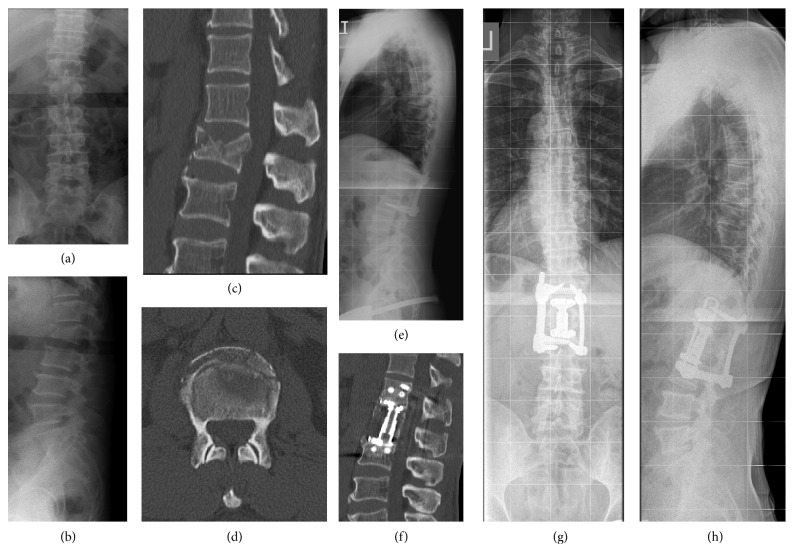
Illustrative case 2. A 51-year-old male patient suffered a L1 fracture during a motor vehicle accident (a, b). The AO Classification was 3.3.1, and the LSC was 9 (c, d). After primary closed reduction and posterior instrumentation at T12–L2 (e), staged anterior surgery using instrumented fusion was conducted after 6 days with partial corpectomy and implantation of a distractible vertebral body replacement. The preoperative RKA and TLA were 20.3° and 9.7°, respectively, and the RKA and TLA at the final follow-up of 33 months postoperatively were 1.4° and 2.2°, respectively (g, h). In the CT scan at the final follow-up, the anterior column was considered fused (f).

The patients in the PA-F group underwent posterior stabilization as described for the POST-I group and underwent staged anterior fusion 4 to 10 days later. Anterior transthoracic fusion was conducted using a video-assisted thoracoscopic approach. After partial corpectomy of the fractured vertebra, either an autologous bone graft or a distractible vertebral body replacement (Synex II, Synthes, Switzerland) was implanted, followed by anterior instrumentation using a rigid screw-plate system (MACS, Braun-Aesculap, Germany) (Figure 2).

Patients were mobilized on postoperative day two without a brace. Postoperative follow-up was scheduled at 6, 12, and 18 months.

Implant removal of the posterior instrumentation was scheduled for all patients after a minimum of 12 months and after obtaining radiographic evidence of bony fracture healing as no posterior spondylodesis and fusion was not conducted.

### 2.2. Radiographic Assessment

The patients underwent preoperative radiographs in the supine position to avoid progressive deformity and neurological symptoms, preoperative CT scans, and full-spine standing biplanar digital radiographs during follow-up. For the patients in the PA-F group, additionally reformatted CT scans of the thoracolumbar junction at follow-up were performed to assess the anterior column fusion. Radiographic images were digitized and studied using imaging software (PACS Magic View VC 42, Rel A, Siemens, Germany).

### 2.3. Radiological Analysis

**Figure 3:**
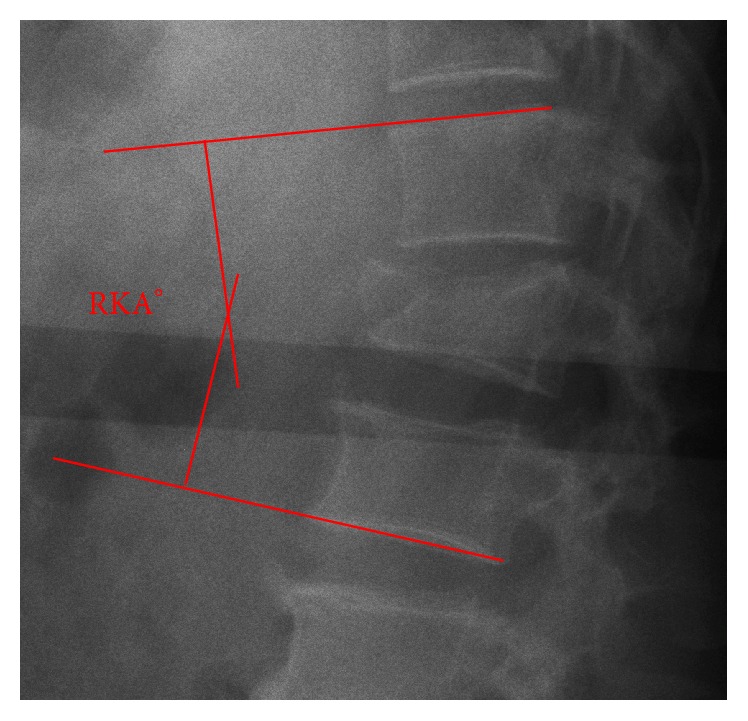
The RKA (regional kyphosis angle) is measured between the tangent of the upper endplate of the cephalad vertebra of the fracture and the tangent of the lower endplate of the caudal vertebra.

The regional kyphosis angle was measured preoperatively (RKA-preop°) and at the final follow-up (RKA-FU°). The measurement technique is illustrated in Figure 3. The thoracolumbar junction angle (TLA°) is defined as the sagittal angle between the endplate tangents of T10 and L2. The TLA was assessed preoperatively and at the final follow-up (TLA-preop/FU°).

**Figure 4:**
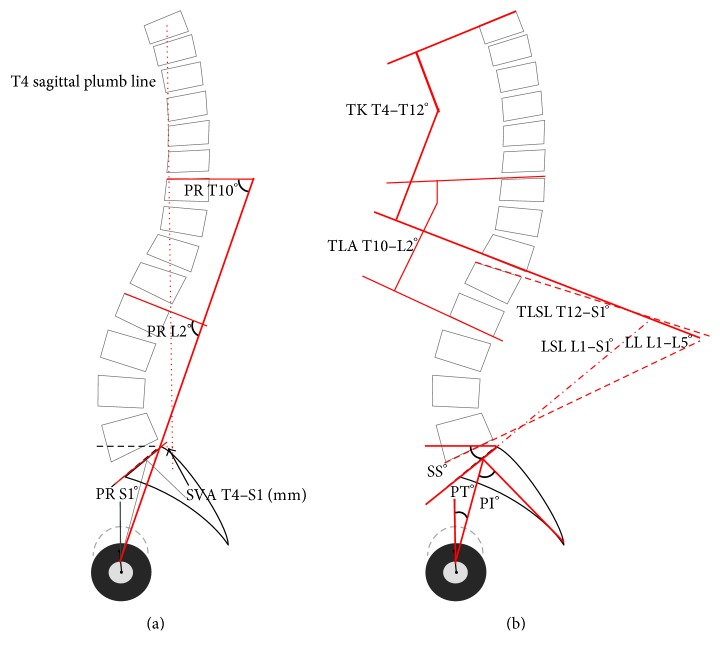
Illustration of the assessed radiographic spinal outcome parameters. RKA: regional kyphosis angle; TLA (T10–L2): thoracolumbar junction angle T10–L2; PI: pelvic incidence; PT: pelvic tilt; SS: sacral slope; TLSL T12–S1: thoracolumbosacral lordosis T12–S1; LSL L1–S1: lumbosacral lordosis L1–S1; LL L1–L5: lumbar lordosis L1–L5; TK T4–T12: thoracic kyphosis T4–T12; PR-S1: pelvic radius to S1; PR-T10: total lumbopelvic lordosis to T10; PR-L2: regional lumbopelvic lordosis to L2; SVA T4–S1: sagittal vertical axis T4 to S1.

The measures of sagittal spinal and spinopelvic balance included thoracolumbosacral lordosis (TLSL T12–S1), lumbar lordosis L1–L5 (LL), lumbosacral lordosis L1–S1 (LSL), thoracic kyphosis T4–T12 (TK), sagittal vertical axis T4-S1 (SVA, mm), pelvic incidence (PI), sacral slope (SS), and pelvic tilt (PT). These measurements are explained elsewhere [32–36]. The spinopelvic alignment was also assessed by measuring the pelvic morphology (PR-S1), total lumbopelvic lordosis (PR-T10), and regional lumbopelvic lordosis (PR-L2) according to Jackson's techniques [37]. The radiographic measurement techniques are shown in Figure 4.

### 2.4. Clinical Outcome Scores

The clinical outcomes were assessed using validated measures, including the Roland Morris Disability Spine Questionnaire (RMDQ) [38], the short-form 36-item questionnaire version 2 (SF-36-v2) with the physical and mental component summary (PCS/MCS), the visual analogue scale (VAS) spine score [39], the Oswestry Disability Index (ODI) [40] (grading was as follows: 0–20%: minimal disability; 21–40%: moderate disability; 41–60%: severe disability; 61–80%: crippled; 81–100%: bed-bound), and the Greenough Low Back Outcome Scale (LBOS) [41, 42]. Statistical analysis for the LBOS was performed by categorizing the results into numeric form as follows: excellent = 1, good = 2, fair = 3, and poor = 4.

### 2.5. Statistical Analysis

The statistical analyses included descriptive statistics, parametric tests (independent two-sided Student's* t*-test and Pearson's correlation coefficient), and nonparametric tests (Wald-Wolfowitz test, Mann–Whitney* U*-test, and Spearman's correlation coefficients). A *p* value less than .05 indicated a statistically significant result. All analyses were carried out using SPSS 11.0 (SPSS Inc., Chicago, USA), Statistica 6.1 (StatSoft, Tulsa, USA), and StatXact (Cytel Software, Cambridge, UK). The statistical analysis was conducted for the overall cohort and subgroups and included an intergroup differential analysis.

## 3. Results

A group of 44 patients, homogenous for sample characteristics, with single-level AO Type 3 burst fractures of L1 or T12 met the inclusion criteria. Among this group, 38 patients (86%) completed the follow-up survey (21 males/15 females). Two patients were excluded because of poor quality of FU radiographs, not enabling accurate assessment of the spinopelvic parameters. A total of 36 patients remained as the study sample for statistical analysis, representing a FU-rate of 81.8%.

Mean patients' age at time of the index procedure was 39 ± 13 years (16–63). The length of follow-up was 69 ± 33 months (18–149). There were 25 patients (69%) with L1 and 11 (31%) with T12 burst fractures of AO Type A3. A total of 22 patients (61%) were treated using posterior instrumentation, and 14 (39%) underwent posteroanterior instrumented fusion.

The most common injury mechanism was a sports-related injury (42%), followed by a fall from height (39%) and a motor vehicle accident (19%). Average hospital stay was 16 ± 7 days. The patient characteristics are summarized in Table 1.

Implant removal was performed in 34 patients after 12 months. In two of the POST-I group patients, the implants were not removed.

### 3.1. Fracture Morphology

AO Type A3.1 fractures accounted for 44% (*n* = 16), A3.2 for 31% (*n* = 11), and A3.3 for 25% (*n* = 9) of these fractures. According to the LSC, there were Type 4 burst fractures in 3, Type 5 in 5, Type 6 in 14, Type 7 in 7, Type 8 in 6 patients, and Type 9 in 1 patient. A total of 78% of patients (*n* = 28) had a LSC > 5. The distribution of fracture subtypes within the overall cohort and among the POST-I and PA-F groups is summarized in Table 2.

### 3.2. Complications

CT scans revealed asymptomatic nonunion of the anterior fusion mass, not requiring further surgical treatment in four patients (29%) in the PA-F-group. None of the patients suffered screw breakage, screw loosening, or construct failure. No major complications occurred postoperatively in either group.

### 3.3. Radiographic Analysis

The detailed results of the radiographic measurements and ranges of the entire sample group, the POST-I group, and the PA-F group are presented in Table 3.

### 3.4. Clinical Outcomes

All of the clinical outcomes for the entire sample group, the POST-I group, and PA-F group are presented in Table 4.

### 3.5. Statistical Analysis

#### 3.5.1. All Patients (*n* = 36)

The baseline characteristics regarding the AO Classification and Load Sharing Classification, preoperative RKA and TLA, and patient characteristics were not significantly different between the POST-I and PA-F groups (*p* > .05).

There was a significant correlation between several radiographic measures at follow-up and the clinical outcome (RKA & SF36-MCS: *p* = .03, *r* = −0.4; LSL & SF36-MCS: *p* = .01, *r* = −0.5; LL & ODI: *p* = .056 (trend), *r* = −0.3).

There were no significant differences between the preoperative and follow-up values for the RKA (11.7 ± 6.6° versus 12.7 ± 9.2°, *p* > .05) and TLA (11.2 ± 5.8° versus 14.7 ± 9.2°, *p* = .08). There was no significant correlation between the preoperative RKA and TLA and the clinical outcome scores (*p* > .05).

The patients' and fracture characteristics did not significantly influence the clinical outcome scores.

### 3.6. Subgroup Analysis

#### 3.6.1. POST-I Group (*n* = 22)

At the follow-up, the SF-36 MCS showed significant inverse correlation with the TLA-FU (*p* = .03, *r* = −0.5) and the TK (*p* = .04, *r* = −0.5). The ODI showed a significant correlation with the TLA-FU (*p* = .02, *r* = 0.5) and inverse correlation with the LL (*p* = .01, *r* = −0.5) and the TK (*p* = .02, *r* = 0.5).

The differences between the preoperative and follow-up RKA and TLA were not significant (*p* > .05).

#### 3.6.2. PA-F Group (*n* = 14)

The sagittal balance according to the SVA had a significant impact on the visual analogue scale (VAS) spine score (*p* = .01, *r* = 0.9). The TK showed significant inverse correlation with the LBOS (*p* = .047, *r* = −0.5) and the ODI (*p* = .02, *r* = −0.6) and correlated with the SF-36 PCS (*p* = .04, *r* = 0.6).

The differences between the preoperative and follow-up RKA and TLA were not significant (*p* > .05).

### 3.7. Intergroup Analysis

Analysis of the differences between the PAF and POST-I group revealed a trend towards significance for the RKA at follow-up (PA-F: 9.6 ± 5.5° versus POST-I: 14.7 ± 10.6°; Δ5.1°, *p* = .07) in favor for the PA-F group. A similar trend existed for the follow-up TLA (PA-F: 12.6 ± 8.7° versus POST-I: 16.0 ± 9.4°; Δ3.4°, *p* = .08).

Regarding the clinical outcomes, no significant differences were observed between the POST-I and PA-F group.

Results of the intergroup analysis are displayed in Tables 3 and 4.

## 4. Discussion

In the current study, the clinical and radiological outcomes following surgical treatment of T12 and L1 burst fractures were evaluated. Strict inclusion criteria enabled a homogenous cohort with an average follow-up of 6 years. Radiological assessment of the spinal and spinopelvic balance parameters was conducted using standing full-spine radiographs at follow-up, and CT scans were used for fusion assessment in the posteroanterior group. To the authors' knowledge, the current study is the first to investigate the clinical long-term outcomes of a homogenous group of patients with isolated thoracolumbar burst fractures (T12 and L1) according to their regional and global sagittal balance.

For the overall sample group, the statistical analysis revealed a significant correlation between the radiographic measures of deformity at follow-up (RKA, LSL, and LL) and several clinical outcome measures. The results indicate that increasing residual deformity of the regional sagittal alignment and alteration of the global sagittal balance can impact clinical outcomes over the long term, even in surgically treated patients. By conducting an outcome analysis of surgically treated fracture patients, our study adds evidence to the increasing data on the impact of sagittal spinal balance on clinical outcomes [5, 26, 27, 32, 33, 43]. Sagittal imbalance caused by posttraumatic deformity and adult degenerative deformity has been shown to promote increasing pain, worse clinical outcomes, and a loss of health-related quality of life [44–46]. In a long-term investigation of the results related to nonsurgical treatment of thoracolumbar burst fractures, Koller et al. [5] demonstrated the impact of long-lasting residual regional deformity and global imbalance on clinical outcomes. In particular, the large number of global, thoracic, and lumbosacral compensatory mechanisms that aim to balance the regional deformity was shown to have a negative impact on clinical outcomes. While the residual kyphotic deformity at the fracture level in our study was smaller than that found in the study by Koller et al. [5], our analysis of the residual deformity revealed that the maintenance of a close-to-physiological sagittal alignment with restoration of the regional kyphosis angle and thoracolumbar junction angle influences the clinical outcome measures.

Currently, two studies have compared the results of combined posteroanterior fusion with posterior-only or anterior-only treatments for thoracolumbar fractures. Both studies failed to show significant differences in the outcome or the superiority of one approach over the other [47, 48]. Notably, the studies did not address the global spinal alignment or the heterogeneity of the sample and fracture characteristics, which may have affected the impact of residual deformity on the clinical outcome. This agrees with the results of the current study where group differences did not achieve statistical significance due to the sample size, although a trend towards a better RKA and TLA at the follow-up in the posteroanterior group compared with the posterior-only group was observed. A larger study sample might have stressed clinical observations of improved maintenance of the posterior reduction and stabilization using the combined posteroanterior approach relative to the posterior-only treatment. The data are in accordance with observations from a multicenter study of the Spine Study Group of the German Association of Trauma Surgery [49]. That study indicated superiority in radiographic deformity reduction using the combined approach compared with the posterior-only approach.

It is worth noting that intergroup analysis revealed no significant differences in the global sagittal spinal and spinopelvic measures or clinical outcome measures when examining 22 and 14 patients in the posterior-only and posteroanterior group, respectively. However, analysis of all patients (*n* = 36) revealed a significant impact of residual regional and global sagittal deformities on the clinical outcomes. These findings are of distinct interest in perspective of a mean follow-up of 6 years and mean patient age of 39 years in our study. It is well documented that with depleted compensatory mechanisms balancing a regional kyphosis over years, symptoms might arise decades later [5, 24].

Previous studies showed loss of the initial reduction in patients who underwent posterior instrumentation only with temporary instrumentation and subsequent implant removal [49–51], particularly in patients with an injured disc adjacent to the burst fracture. Accordingly, postoperative measures of reduction and deformity were not included in the current study, focusing on sagittal spinal and spinopelvic measures at follow-up in a sample of fracture patients treated surgically and on the impact residual deformity had on the clinical outcomes at mid- to long-term follow-up.

## 5. Conclusion

The treatment of thoracolumbar fractures without neurological deficits remains controversial because most studies have evaluated heterogeneous patient samples, fracture patterns, and vertebral levels with various follow-up lengths. Therefore, it is difficult to compare the present study with previous studies. We comprehensively evaluated the association between regional and global spinal alignment and long-term outcomes in surgically treated “pure” thoracolumbar fractures of T12 or L1 only with strict inclusion criteria to obtain a homogeneous study cohort. The results demonstrate the interdependency between sagittal alignment and clinical outcomes. They also support the assumption that stable restoration of thoracolumbar alignment towards normalcy and its maintenance has a positive impact on the clinical outcome.

To elucidate the ideal surgical treatment for each individual burst fracture subtype, further research is needed with larger samples and detailed assessments of fracture characteristics in perspective of global spinal alignment and compensatory mechanisms. The current study serves as a predecessor for such studies.

## Figures and Tables

**Table 1:** Demographics and patients' characteristics.

	All patients (*n* = 36)	POST-I group (*n* = 22)	PA-F group (*n* = 14)	Intergroup analysis level of significance
Age (years)	39 ± 14 years (16–63)	42 ± 14 years (20–63)	34 ± 10.6 years (19–55)	*p* > .05
Follow-up (months)	69 ± 33 months (17–149)	66 ± 37 months (17–149)	74 ± 26 months (24–105)	*p* > .05
Male	58.3% (*n* = 21)	50% (*n* = 11)	71.4% (*n* = 10)	*p* > .05
Female	41.7% (*n* = 15)	50% (*n* = 11)	28.6% (*n* = 4)	*p* > .05
*Fracture level*				
T12	30.6% (*n* = 11)	27.3% (*n* = 6)	35.7% (*n* = 5)	*p* > .05
L1	69.4% (*n* = 25)	72.7% (*n* = 16)	64.3% (*n* = 9)	*p* > .05

**Table 2:** Fracture characteristics and distribution of fracture types: There was no significant difference in the distribution of fracture subtypes between POST-I and PAF group (*p* > .05).

	AO Classification				Load Sharing Classification					
Subtype	A3.1.1	A3.1.2	A3.2.1	A3.3.1	4	5	6	7	8	9
All patients (*n* = 36)	15 (41.7%)	1 (2.8%)	11 (30.6%)	9 (25%)	3 (8.3%)	5 (13.9%)	14 (38.9%)	7 (19.4%)	6 (16.7%)	1 (2.8%)
POST-I group (*n* = 22)	11 (50%)	1 (4.55%)	6 (27.3%)	4 (18.2%)	3 (13.6%)	3 (13.6%)	6 (27.3%)	6 (27.3%)	4 (18.2%)	0
PA-F group (*n* = 14)	4 (28.6%)	0	5 (35.7%)	5 (35.7%)	0	2 (14.3%)	8 (57.1%)	1 (7.1%)	2 (14.3%)	1 (7.1%)

**Table 3:** Radiographic measurement outcomes.

	All patients	POST-I group	PA-F group	Intergroup analysis level of significance
	(*n* = 36)	(*n* = 22)	(*n* = 14)	
	mean ± SD	mean ± SD	mean ± SD	
	(range)	(range)	(range)	
*Radiographic parameters*				
RKA preop°	11.7 ± 6.6	11.1 ± 6.5	12.6 ± 6.8	*p* > .5
	(2–25)	(2–25)	(2.5–22.6)	
RKA at follow-up°	12.7 ± 9.2	14.7 ± 10.6	9.6 ± 5.5	*p* = .07
	(0.8–35.8)	(2.3–35.8)	(0.8–16.1)	
TLA (T10–L2) preop°	11.2 ± 5.8	11.7 ± 5.8	10.3 ± 5.8	*p* > .5
	(0.2–21.8)	(0.2–21.8)	(2–20.3)	
TLA (T10–L2)	14.7 ± 9.2	16 ± 9.5	12.6 ± 8.8	*p* = .08
at follow-up°	(1.8–37.1)	(2.2–37.1)	(1.8–27.2)	
*Spinal and spinopelvic parameters at follow-up*				
PI°	53.1 ± 9.8	53.9 ± 9.5	51.7 ± 10.5	*p* > .5
	(31.5–74.7)	(39.7–74.7)	(31.5–68)	
PT°	17.2 ± 81.4	18.2 ± 7.8	15.7 ± 8.7	*p* > .5
	(2.3–32)	(6.7–32)	(2.3–29.2)	
SS°	36.6 ± 7.8	34.7 ± 6.1	39.9 ± 9.4	*p* > .5
	(27.2–60.4)	(27.2–50.2)	(29.6–60.4)	
TLSL T12–S1°	−49.7 ± 10.1	−48.1 ± 9.6	−52.5 ± 10.7	*p* > .5
	(−65.1–(25.9))	(−62.7–(−25.9))	(−65.1–(−28.9))	
LSL L1–S1°	−50 ± 20.7	−49.9 ± 12.3	−50.1 ± 30.4	*p* > .5
	(−70.5–47.2)	(−66.9–(−20.5))	(−70.5–47.2)	
LL L1–L5°	−42.5 ± 11.3	−42.5 ± 13.4	−42.4 ± 7.1	*p* > .5
	(−58.3–(−11.4))	(−58.3–(−11.4))	(−53–(−26))	
TK T4–T12°	36.5 ± 15.7	33.4 ± 13.9	40.7 ± 17.4	*p* > .5
	(1.8–77.5)	(1.8–58.6)	(2–77.5)	
PR-S1°	−30.3 ± 16.1	−31.8 ± 8.1	−27.9 ± 24.3	*p* > .5
	(−52.1–40)	(−47.2–(−14.6))	(−52.1–40)	
PR-T10°	−59.9 ± 43.9	−50.7 ± 53.9	−74.4 ± 11.5	*p* > .5
	(−93.5–77.6)	(−93.5–77.6)	(−89.9–(−45.5))	
PR-L2°	−84.2 ± 8	−84.5 ± 7.2	−83.6 ± 9.4	*p* > .5
	(−97.4–(−64.1))	(−95.2–(−66.8))	(−97.4–(−64.1))	
SVA T4–S1 (mm)	−22.2 ± 17.7	−21.7 ± 19.2	−22.9 ± 15.9	*p* > .5
	(−54.8–13.9)	(−54.8–13.9)	(−51.3–0)	

RKA: regional kyphosis angle; TLA (T10–L2): thoracolumbar junction angle T10–L2; PI: pelvic incidence; PT: pelvic tilt; SS: sacral slope; TLSL T12–S1: thoracolumbosacral lordosis T12–S1; LSL L1–S1: lumbosacral lordosis L1–S1; LL L1–L5: lumbar lordosis L1–L5; TK T4–T12: thoracic kyphosis T4–T12; PR-S1: pelvic radius to S1; PR-T10: total lumbopelvic lordosis to T10; PR-L2: regional lumbopelvic lordosis to L2; SVA T4–S1: sagittal vertical axis T4 to S1.

**Table 4:** Results of clinical outcome scores.

	All	POST-I group	PA-F group	Intergroup analysis level of significance
	(*n* = 36)	(*n* = 22)	(*n* = 14)	
RMDQ	3.8 ± 4.9	3.3 ± 4.2	4.6 ± 6.0	*p* > .5
	(0–21)	(0–14)	(0–21)	
LBOS	2.3 ± 1.0	2.1 ± 1.1	2.6 ± 0.7	*p* > .5
	(1–4)	(1–4)	(2–4)	
ODI%	17 ± 17%	16.3 ± 17.1%	20 ± 20%	*p* > .5
	(0–67%)	(0–67%)	(0–60%)	
VAS	21.8 ± 22.3	17.1 ± 18.2	32.1 ± 27.8	*p* > .5
	(0–83.4)	(0–57.6)	(0–83.4)	
SF-36 PCS	48.1 ± 9.3	49.3 ± 9.4	46.1 ± 14.3	*p* > .5
	(24–61)	(30.2–61)	(24–57.3)	
SF-36 MCS	49 ± 14.2	51 ± 14.1	45.7 ± 14.3	*p* > .5
	(10.6–66.8)	(10.6–66.8)	(21.6–61.6)	

RMDQ: Roland Morris Disability Spine Questionnaire; LBOS: Greenough's Low Back Outcome Scale; ODI: Oswestry Disability Index; VAS: visual analogue scale specific to the spine; SF-36 PCS: short-form 36-item questionnaire physical component summary; SF-36 MCS: short-form 36-item questionnaire mental component summary.
